# Direct Measurements of Human Colon Crypt Stem Cell Niche Genetic Fidelity: The Role of Chance in Non-Darwinian Mutation Selection

**DOI:** 10.3389/fonc.2013.00264

**Published:** 2013-10-14

**Authors:** Haeyoun Kang, Darryl Shibata

**Affiliations:** ^1^Department of Pathology, CHA Bundang Medical Center, CHA University, Seongnam-si, South Korea; ^2^Department of Pathology, University of Southern California Keck School of Medicine, Los Angeles, CA, USA

**Keywords:** stem cell niche, non-Darwinian, neutral drift, human, replication errors, aging, Muller’s ratchet

## Abstract

Perfect human stem cell genetic fidelity would prevent aging and cancer. However, perfection would be difficult to achieve, and aging is universal and cancers common. A hypothesis is that because mutations are inevitable over a human lifetime, downstream mechanisms have evolved to manage the deleterious effects of beneficial and lethal mutations. In the colon, a crypt stem cell architecture reduces the number of mitotic cells at risk for mutation accumulation, and multiple niche stem cells ensure that a lethal mutation within any single stem cell does not lead to crypt death. In addition, the architecture of the colon crypt stem cell niche may harness probability or chance to randomly discard many beneficial mutations that might lead to cancer. An analysis of somatic chromosome copy number alterations (CNAs) reveals a lack of perfect fidelity in individual normal human crypts, with age-related increases and higher frequencies in ulcerative colitis, a proliferative, inflammatory disease. The age-related increase in somatic CNAs appears consistent with relatively normal replication error and cell division rates. Surprisingly, and similar to point mutations in cancer genomes, the types of crypt mutations were more consistent with random fixation rather than selection. In theory, a simple “non-Darwinian” way to nullify selection is to reduce the size of the reproducing population. Fates are more determined by chance rather than selection in very small populations, and therefore selection may be minimized within small crypt niches. The desired effect is that many beneficial mutations that might lead to cancer are randomly lost by drift rather than fixed by selection. The subdivision of the colon into multiple very small stem cell niches may trade Darwinian evolution for non-Darwinian somatic cell evolution, capitulating to aging but reducing cancer risks.

## Introduction

**Figure 1:**
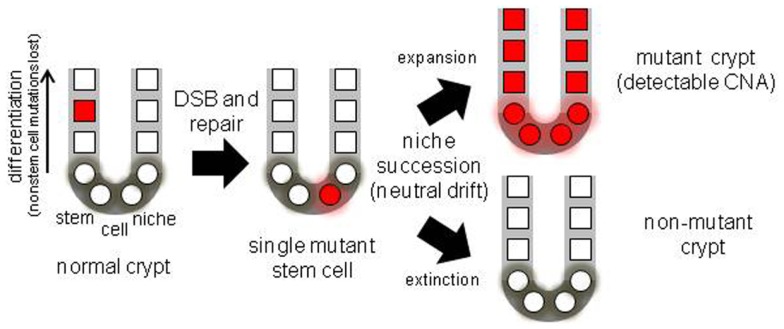
**Diagram of a colon crypt stem cell niche, illustrating niche stem cell turnover**. With multiple niche stem cells, a mutation will become detectable if its stem cell eventually dominates its niche.

The mechanisms responsible for human stem cell genetic fidelity are difficult to directly study because of the impracticality of experimental manipulations. However, the numbers and types of somatic mutations accumulated during a lifetime are end measures of stem cell genetic fidelity. It should be possible to infer from mutations the various mechanisms that may help limit both the accumulation and phenotypic consequences of human somatic mutations. Mutations can lead to cancer and aging, and replication errors are major potential sources of somatic alterations. The colon is highly mitotic organ, with most epithelial cells replaced weekly (1, 2). The colon is subdivided into millions of small clonal units called crypts. Human colon crypts are small (∼2,000 cells) glands maintained by multiple stem cells and a stem cell hierarchy (Figure 1). A stem cell hierarchy helps limit mutation accumulation because only a small fraction of all cells (∼5%) in the crypt are stem cells. Mutations in non-stem cells normally cannot accumulate because these cells are lost within a week. Within stem cells, a primary defense against mutations is DNA replication fidelity, with the immortal strand hypothesis (3) an extreme scenario where any replication errors are subsequently lost because newly synthesized DNA strands are asymmetrically distributed to non-stem cell daughters. Another mechanism of mutation avoidance is stem cell quiescence, where stem cells divide infrequently. Stem cells may also be extremely sensitive to DNA damage, and therefore mutant stem cells could be eliminated by apoptosis (4).

Recent mouse studies illustrate that Lgr5+ intestinal stem cells are not quiescent, with mitotic rates estimated at once per day (5). However, there may be many different types of intestinal stem cells, and potentially some of these are more quiescent (6). The immortal strand hypothesis (3) in intestinal crypts is controversial, with evidence for and against asymmetric DNA strand segregation [see for example references (7–12)].

Human crypt stem cell studies are limited because of the inability to use the powerful fate mapping experimental techniques of model systems. However, humans are long-lived and therefore it is possible to measure the “success” of fidelity mechanism by directly measuring mutations in colons isolated from different aged individuals (13). Measurable mutations should accumulate over decades of aging, even with relatively low mutation or stem cell division rates. Human stem cell fidelity mechanisms can be inferred from the numbers and types of somatic mutations accumulated over a lifetime.

A practical problem with measuring somatic mutations is that because different mutations occur in different cells, the frequency of any specific mutation in a population of human cells is likely to be too low (<5%) to measure with most techniques. Crypts contain multiple stem cells (2, 6) and a mutation in one stem cell would be masked by surrounding non-mutant cells in the same crypt. However, progeny of a single mutant stem cell may expand and fill the crypt because stem cells are extrinsically defined by a surrounding niche, and their survival is probabilistic. Niche stem cells usually divide asymmetrically to produce one stem and one non-stem cell daughter, but may also divide symmetrically to produce two stem cell daughters (expansion) or two non-stem cell daughters (extinction). The total number of niche stem cells remains constant, but eventually all stem cell lineages except one become extinct (Figure 1) by a niche succession process called neutral drift (14, 15). Crypt niche stem cell neutral drift has been characterized in mice using fate marking strategies that are impractical in humans. However, measurements of passenger methylation patterns in human crypts are also consistent with multiple stem cells per crypt and neutral drift, with niche succession intervals estimated at about 8 years (16). This human crypt niche succession time estimate is uncertain because of a paucity of experimental opportunities to characterize human crypt niche dynamics. Another study also found that crypt stem cell succession times are longer in humans compared to mice (17).

A mutant crypt stem cell can either suffer extinction or expand to fill the entire niche (fixation), and become detectable (Figure 1). Neutral drift appears to constantly recur in the absence of mutations (14, 15), but potentially a mutation may influence this process by conferring positive or negative selection to its cells. Therefore, crypt mutation frequencies reflect many aspects of stem cell genetic fidelity, from replication fidelity to the ultimate fates of mutant stem cell progeny. Here data (13) from crypt genomes scanned with high density SNP microarrays for chromosomal copy number alterations (CNAs) from individuals of different ages are further analyzed.

## Materials and Methods

### Colon crypts and analysis

The data are from Ref. (13), using the “Reference” method with additional analysis (see below). Briefly, single individual whole normal crypts were obtained from ∼1 cm^2^ mucosal patches of fresh colectomies at the University of Southern California Keck School of Medicine using an EDTA washout method (16). Procurement of the excess tissue was approved by the Institutional Review Board at the University of Southern California. Normal crypts were isolated from normal appearing colon obtained at least 10 cm away from a tumor. DNA was extracted in 15 μl of TE with 1 μl of 20 mg/ml Proteinase K at 56°C for 4 h followed by boiling for 7 min. All this DNA was used for the SNP microarrays (610-Quad, 660-Quad, 730-OmniExpress) using standard Illumina protocols.

Data were processed using GenomeStudio with a quality threshold of 0.15. Call rates for 180 crypts from 18 colons were variable (49.5–99.8%, average 89.5%), likely because each crypt has ∼20 ng of DNA, versus the recommended 200 ng of DNA per microarray. Crypts with call rates greater than 60% (*N* = 176) were further analyzed. Multiple crypts from the same colon were compared pairwise at the SNPs. The genotype of a “reference” crypt (typically one with a higher call rate) was determined with GenomeStudio. The reference data were filtered by eliminating no call SNPs and the homozygous (AA, BB) loci. The filtered reference crypt data were compared pairwise with the subject crypt. A likely CNA was identified by loss of heterozygosity (LOH) in the subject crypt at a string of adjacent heterozygous SNPs. At least two different reference crypts were used.

The percentages of SNPs with LOH outside of these likely CNAs were less than 2% of the AB SNPs, even with lower call rates. Most of these non-CNA LOH SNPs were singletons, with fewer longer strings. The probabilities that LOH occurred by chance in the likely CNAs were calculated using the Poisson distribution, with error probabilities estimated from non-CNA LOH SNP frequencies. The smallest CNA had five adjacent heterozygous SNPs and the probabilities that the CNAs < 1 Mb occurred by chance were all less than 0.01 and typically much smaller. All CNAs were verified by manual inspection.

Log R ratios were used to distinguish between deletions or gene conversion (GC). A deletion was called when the average log R ratio of the LOH region was more than 0.25 lower than its flanking regions without LOH. Duplications were not formally analyzed for because of the wide variations in log R ratios, but were visually identified as large regions with three chromosome copies with increased log R ratios.

### Additional analysis

The crypt SNP microarray data were further reanalyzed with Nexus Copy Number software (Version 7, BioDiscovery, Hawthorne, CA, USA) using the SNP-FASST2 analysis with default settings and a paired crypt analysis. The software did not identify all CNAs identified in Ref. (13), but all of the previously identified CNAs were evident with manual inspection. One additional deletion on chromosome 1q in an 85-year-old male was identified by the software, which was missed by the previous analysis because the mutant cells were a minority of all cells, with heterozygous SNPs still called AB by GenomeStudio.

The proportions of mutant cells within a crypt containing a deletion were estimated by comparing average BAFs of the subject crypt that were homozygous (BAF_AA_ or BAF_BB_) in the reference crypt, versus SNPs that became homozygous (BAF_aa_ or BAF_bb_). Simplistically, if all cells within a crypt contained the deletion, then BAFs at the previous AB SNPs would be identical to the SNPs that were always AA or BB. The formula used was,

$$P=1-\left[\frac{\frac{\left({\text{BAF}}_{\text{AA}}-{\text{BAF}}_{\text{aa}}\right)}{\left(0.5-{\text{BAF}}_{\text{AA}}\right)}+\frac{\left({\text{BAF}}_{\text{BB}}{\text{ - BAF}}_{\text{bb}}\right)}{\left({\text{BAF}}_{\text{BB}}-0.5\right)}}{2}\right].$$

## Results

### Chromosome copy number alterations increase with age

**Figure 2:**
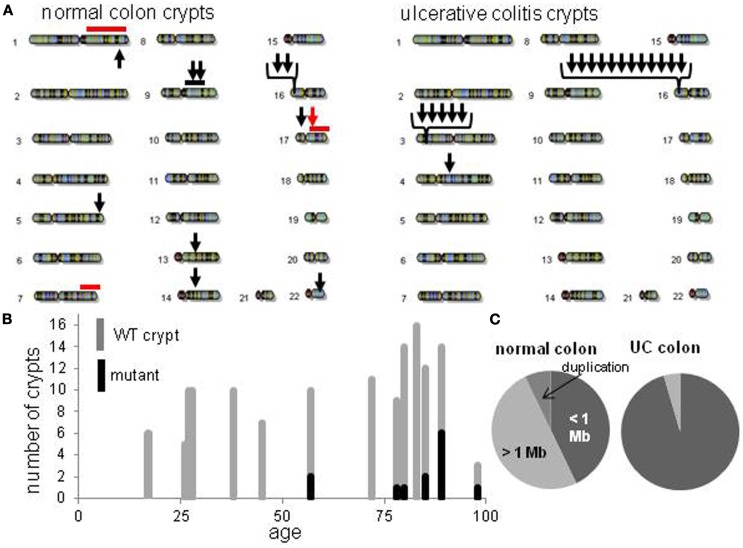
**Crypt chromosome copy number alterations (CNAs) across all non-UC and UC crypts**. **(A)** Locations of chromosome CNAs. Arrows indicate loss of heterozygosity, red bars indicate gene conversion, black bar is a duplication. **(B)** Increase in mutant non-UC crypts with age. **(C)** Relative proportions of chromosome CNA types in normal colon crypts and normal UC crypts.

Detectable human crypt CNAs increased with age (Table 1 and Figure 2). No CNAs were found in 48 crypts from individuals less than 50 years old, with 14 CNAs in 13 of 89 crypts (15%) from individuals greater than 50 years of age (*p* = 0.0042, Fisher’s exact test). Call rates were not significantly different between crypts with and without CNAs. CNAs sizes (Table 2) ranged from ∼10,000 bp (LOH at five adjacent SNPS) to 98 Mb (LOH at 3,996 SNPs). Only one crypt had more than one CNA. Seven CNAs were greater than 1 Mb, and six were smaller than 1 Mb (Figure 2B). A single 44 Mb interstitial duplication was detected. The CNAs appeared to be in the majority (average 93%, range 34–100%) of cells within each crypt (Table 2). Only one LOH region was estimated to be in less than 80% of the crypt cells. Therefore, most of the CNAs were fixed or near fixations in their crypts.

**Table 1:** **Colon crypt chromosome copy number alterations**.

Individual	Age/sex	Disease	Crypts	Mutant crypts (%)	Total CNAs
**NORMAL**					
1	17/M	CRC	6	0 (0)	0
2	26/F	CRC	5	0 (0)	0
3	27/F	Diverticulitis	10	0 (0)	0
4	28/F	CRC	10	0 (0)	0
5	36/F	Endometriosis	10	0 (0)	0
6	45/M	CRC	7	0 (0)	0
7	57/M	CRC	10	2 (20)	2
8	72/M	CRC	11	0 (0)	0
9	78/F	Diverticulitis	9	1 (11)	1
10	80/M	CRC	14	1 (7)	1
11	83/M	CRC	16	0 (0)	0
12	85/M	CRC	12	2 (17)	2
13	89/F	CRC	14	6 (43)	7
14	98/M	CRC	3	1 (33)	1
Total			137	13 (9)	14
	<50 years		48	0 (0)	0
	>50 years		89	13 (15)	14
**COLITIS**					
15	30/M	UC	12	6 (50)	8
16	56/M	UC	11	7 (64)	12
17	57/F	UC	8	1 (12)	1
18	46/F	UC	7	1 (14)	1
Total			38	15 (39)	22

CRC, colorectal cancer; UC, ulcerative colitis.

**Table 2:** **Chromosome copy number alteration characteristics**.

Crypt	Age	Location^a^	Size (Mb)	Type	Mutant (%)
**NORMAL**					
7a	57	13q14.11	0.73	Deletion	83
7b	57	17q12	0.015	Deletion	94
9a	78	7q33–q44	24	GC (arm loss)	98
10a	80	1q12–q44	99	GC (arm loss)	96
12a	85	17q12–q25.3	46	GC (arm loss)	99
12b	85	1q43	2.4	Deletion	34
13a	89	5q35.1	3.6	Deletion	95
		9q12–q31.2	44	Duplication	NC
13b	89	9q21.13	0.23	Deletion	89
13c	89	16p13.3	0.073	Deletion	97
13d	89	16p13.3	0.27	Deletion	90
13e	89	17p12	1.1	Deletion	98
13f	89	22q13.1	0.26	Deletion	96
14a	98	9q22.32	3.3	Deletion	99
**COLITIS**					
15a	30	16p13.3	0.38	Deletion	96
15b	30	16p13.3	0.41	Deletion	95
15c	30	16p13.3	0.34	Deletion	86
15d	30	3p14.2	0.082	Deletion	96
		3p14.2	0.13	Deletion	96
		16p13.3^a^	0.35	Deletion	89
15e	30	16p13.3^a^	0.35	Deletion	91
15f	30	3p14.2	0.075	Deletion	98
16a	56	3p14.2	0.14	Deletion	100
16b	56	16p13.3	0.21	Deletion	96
16c	56	16p13.3	0.60	Deletion	97
16d	56	16p13.3	0.20	Deletion (HD)	NC
		16p13.3^a^	0.15	Deletion (HD)	NC
16e	56	16p13.3	0.010	Deletion	98
		16p13.3	0.11	Deletion	98
16f	56	3p14.2	0.11	Deletion	100
		16p13.3	0.44	Deletion (HD)	NC
		16p13.3^a^	0.24	Deletion (HD)	NC
16g	56	4q21.1–q22.1	12	Deletion	94
		16p13.3	0.19	Deletion	96
17a	57	16p13.3	0.32	Deletion	98
18a	46	3p14.2	0.042	Deletion	95

^a^ Other 16p allele within the same colon.

NC, not calculated.

The CNAs were deletions, GC events, and one duplication (Table 2). Log R ratios were consistent with LOH by GC for the three largest CNAs that involved nearly the whole 1q chromosome arm (98 Mb) and the distal ends of 17q (46 Mb) and 7q (24 Mb). These large CNAs included their telomeres, and could be generated by a single double strand DNA break (DSB). The ten other CNAs with reduced log R ratios appeared to be simple deletions generated by two DSBs. The single interstitial duplication would also require at least two DSBs.

### Different types of chromosome copy number alterations in inflammatory bowel disease

Normal colon crypts (*N* = 36) were also analyzed from four patients with ulcerative colitis (UC), which is characterized by inflammation and regeneration (18). There were more UC CNAs with 39% of crypts having CNAs (Tables 1 and 2). Five crypts had two or more different CNAs. All UC CNAs appeared to be deletions (decreased log R ratios), and were present in more than 85% of the crypt cells. Call rates were not significantly different between UC crypts with and without CNAs.

The UC CNAs were different from the CNAs in non-UC crypts (Figures 2A,C). A single large deletion (∼12.4 Mb) was found in one UC crypt, but the 21 other UC CNAs were small (<1.0 Mb) non-identical deletions clustered at two “hotspots” at 3p14.2 and 16p13.3. Four UC crypts had multiple 3p14.2 or 16p13.3 deletions, some on both the maternal and paternal alleles, resulting in homozygous deletions (HD) in two crypts. The 3p14.2 deletion (*N* = 6) was present in the FHIT locus, a known DNA fragile site (19). The 16p13.3 deletion was more common (*N* = 15), and was also observed twice in the non-UC colon from patient 13. The 16p13.3 deletion is in a region commonly deleted in cancer cell lines (“16p 6 Mb unexplained”), that also appears to be a DNA fragile site (20). Therefore, DSBs at two specific DNA fragile sites are common in normal UC crypts.

### Expected chromosome copy number frequencies with age

**Figure 3:**
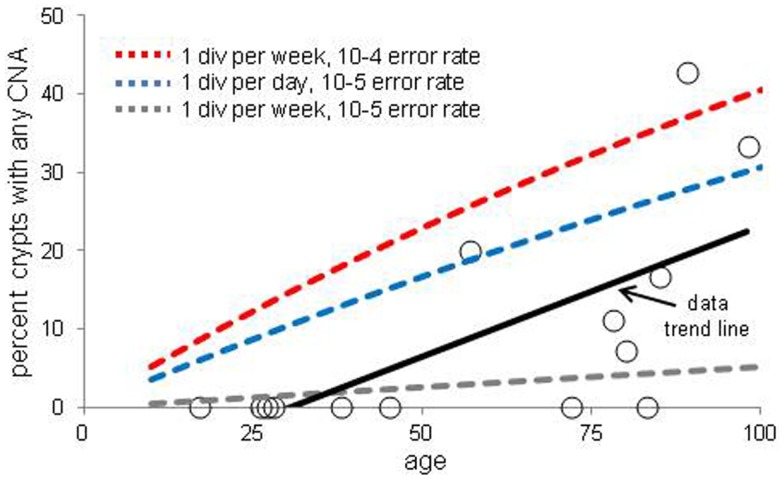
**Percent of crypts with any chromosome copy number alteration versus age**. Circles are averaged experimental data and the dotted lines indicate calculated mutation frequencies with different combinations of mutation and stem cell division rates. A regression analysis indicates that the experimental increase in CNA frequency with age is significant (*p* = 0.01). The calculated mutation frequencies do not account for the lag between a mutation in a stem cell and the time needed to become detectable by niche succession.

The increases in crypt CNAs with aging may reflect abnormal losses of stem cell fidelity or could represent mutation frequencies expected with normal mutation and cell division rates. It is possible to calculate how CNA frequencies should increase with age if one knows the normal error and division rates of crypt stem cells. The CNA mutation rate in normal cells is uncertain but chromosomal instability (CIN) of ∼0.001 chromosomal changes per division have been measured in colorectal cancer (CRC) cell lines (21, 22). Assuming replication fidelity is ∼10–100X higher in normal cells (23–25), stem cell division every day to once a week, and a single stem cell per crypt, expected frequencies of mutant crypts with age were plotted (Figure 3). With this limited data, observed mutant crypt frequencies are not markedly different from that expected with these relatively modest combinations of mutation and division rates. The modeled increase in mutant crypts is roughly linear with age, and a lag between the acquisition of a CNA in a single stem cell and its subsequent fixation to detectable levels in its niche (estimated at ∼8 years in human crypts (16) can help account for the relative lack of mutant crypts at earlier ages.

### Lack of evidence for mutation selection in crypt stem cell niches

**Figure 4:**
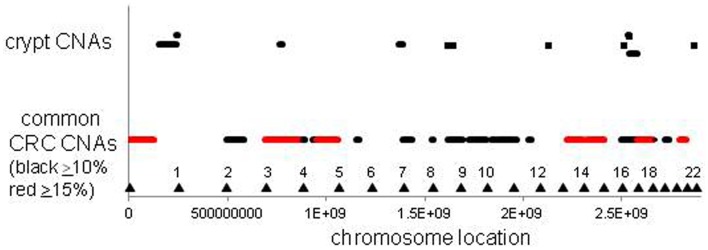
**Relative locations of crypt LOH chromosome copy number alterations and common CRC LOH copy number alterations [from Ref. (26)]**. The locations of the crypt CNAs did not preferentially fall within common CRC CNA intervals, but appeared randomly scattered by chance (see Table 3). Triangles indicate chromosome ends.

The CNAs in normal crypts could reflect selection for mutations that confer selective advantages, or random mutations fixed due to neutral drift. Although it is uncertain which CNAs confer selective advantages to a crypt stem cell, CNAs commonly found in CRCs may predispose to neoplastic progression. We therefore compared the chromosomal locations of the 12 crypt LOH regions (non-fragile sites) to 41 common LOH regions found in 269 MSI negative CRCs from Ref 26 (Figure 4). Similar to the non-UC colon crypts, most CRC LOH regions (78%) were greater than 1 Mb. The regions commonly deleted in CRCs include much of the genome (∼45% of the genome for ≥10% mutation frequencies, and ∼33% for ≥15% mutation frequencies), and the crypt CNAs may fall within these regions by chance. Of the 12 crypt LOH CNAs, 7 overlapped with CRC LOH CNAs with mutation frequencies ≥10%, but only 2 overlapped with CRC mutation frequencies ≥15% (Figure 4). To test whether crypt CNAs were over or under-represented within the CRC CNA regions, a Chi-square test was performed (Table 3). The observed crypt CNA locations with respect to the CRC CNAs were not significantly different than expected by chance (*p* > 0.05).

**Table 3:** **Crypt chromosome copy number alterations versus common colorectal chromosome copy number alterations**.

	CRC LOH 10% frequency, *p* = 0.353*		CRC LOH 15% frequency, *p* = 0.231	
	Observed	Expected^a^	Observed	Expected^b^
Crypt LOH in CRC LOH intervals	7	5.4	2	4
Crypt LOH outside CRC LOH intervals	5	6.6	10	8

*Two tailed *p* value, Chi-square.

^a^ CRC LOH regions (≥10% mutation frequencies) cover ∼45% of genome.

^b^ CRC LOH regions (≥15% mutation frequencies) cover about ∼33% of genome.

Another study measured CNAs in normal whole human blood cells and also found age-related increases, with ∼2–3% detectable CNA incidence in the elderly (27). Blood cells are mixtures of many different cell types that originate from hematopoietic stem cells in multiple widespread bone marrow niches (28). The detection of a CNA in the blood implies the spread of a mutant hematopoietic stem cell to multiple bone marrow niches. In contrast to the colon crypt CNAs that arise within isolated single small niches, the whole blood CNAs were commonly found within chromosomal regions frequently altered in hematopoietic malignancies and normal individuals with detectable blood CNAs had higher risks for subsequent hematopoietic malignancies.

## Discussion

The numbers and types of somatic mutations accumulated over a lifetime reflect different aspects of stem cell fidelity. Mutations are potentially deleterious, and in theory, there may exist special mechanisms that prevent their accumulation within stem cell lineages (3). Primary defenses against somatic mutations are high replication fidelity, efficient DNA repair, and reduced stem cell divisions. The current data indicate that more than 10% of normal human crypts accumulate at least one measurable CNA after the age of 50 years. As illustrated in Figure 3, the observed CNA accumulation is consistent with relatively high replication fidelity and low stem cell division rates. Therefore, even with high genetic fidelity, somatic mutations can accumulate because of long human lifetimes. Other studies using histologic markers have also demonstrated age-related increases in human crypt mutations (29, 30).

Potentially the observed CNAs may have occurred secondary to losses of normal stem cell fidelity. A cell with increased genetic instability would be expected to accumulate multiple mutations. For example, CRC genomes typically have multiple CNAs (25, 26). However, generally only a single CNA was found in each crypt, which is more consistent with random mutation rather than a crypt specific decrease in genetic fidelity.

### Lack of stem cell environmental buffering

Stem cell genomes may be protected from environmental stresses. However, the increased CNA frequencies in UC crypts illustrate that the local microenvironment can influence stem cell genetic fidelity. UC is characterized by severe inflammation with tissue damage and regeneration (18). Potentially increased cell proliferation would simply result in “accelerated” stem cell aging, with more but the same types of CNAs observed in older non-UC crypts. The current data indicate a distinct UC CNA signature characterized by high frequency small (<1 Mb) deletions at two specific DNA fragile sites. Fragile site deletions likely reflect replication stress (31), which is consistent with the higher proliferation of UC. There are multiple known human DNA fragile sites (31) and it is uncertain why only two such sites were commonly altered in normal UC crypts. This distinct mutation signature illustrates that stem cell genomes are sensitive to their microenvironments, and that UC may increase cancer risks by increasing the numbers of specific mutation types.

### Stem cell architecture: managing mutations by playing dice

Stem cell genetic fidelity is high but appears insufficient to protect against detectable mutation accumulation or chronic environmental stresses, especially over decades. Given that mistakes are inevitable, other downstream mechanisms may minimize the unwanted consequences of either deleterious or beneficial mutations. Such a secondary line of defense is the probabilistic nature of a stem cell niche architecture. Stem cells are a fraction (∼5%) of all crypt cells, so most replication errors occur in non-stem cells and are lost. More importantly, there are multiple stem cells per crypt that are extrinsically defined by a niche. These stem cells normally turnover such that eventually progeny of only one current stem cell occupy the entire niche (Figure 1). This stem cell turnover is an important downstream mechanism for managing genetic infidelity. An unwanted consequence of lethal mutations is tissue loss. A crypt maintained by a single stem cell would be extremely vulnerable to lethal mutations. A crypt maintained by multiple immortal stem cells that always divided asymmetrically would also lack a mechanism to compensate for the death of its stem cells. Multiple niche stem cells protect the crypt against the deleterious effects of lethal mutations because the loss of any stem cell is readily compensated by the expansion (symmetrical division) of a neighboring stem cell lineage.

An important question is how the dominant niche stem cell is chosen. In the absence of mutation, all niche stem cells are similar, and episodic succession occurs through neutral drift (14, 15). With mutation, selection could have both desirable and undesirable consequences. Stem cells with non-lethal deleterious mutations would be eliminated by purifying selection, which would mitigate aging. However, stem cells with beneficial mutations would become dominant, which could predispose to tumorigenesis.

**Figure 5:**
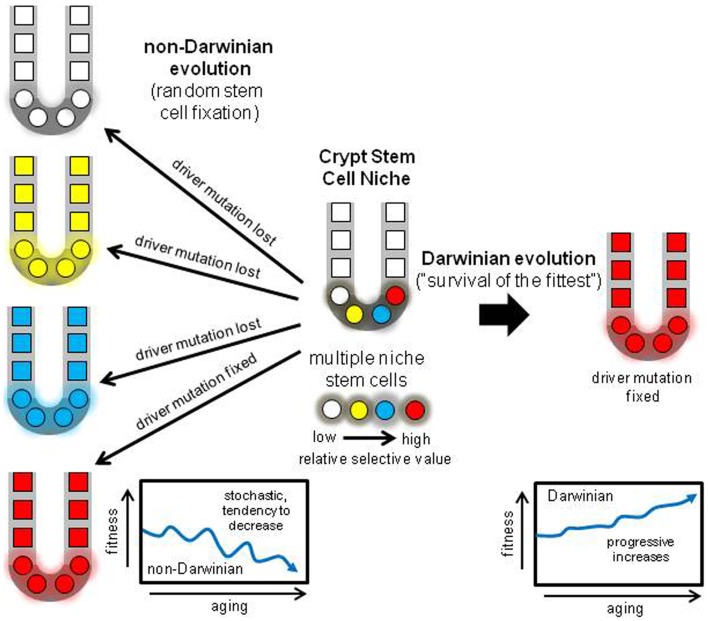
**Darwinian versus non-Darwinian stem cell niche evolution**. Mutations may increase or decrease cell fitness. With multiple stem cells subject to selection, progeny with the highest fitness should reliably dominate the niche, paradoxically increasing fitness with age and predisposing to cancer. With the non-Darwinian evolution favored by very small niche populations, chance or drift more determines niche succession, and almost any stem cell may become fixed, even stem cells with lower relative fitness. The result is the random loss of many driver mutations, and more consistent with aging, a stochastic tendency for decreased crypt fitness.

A niche with multiple neighboring stem cells might appear to be an ideal Darwinian setting to discriminate between even minor fitness differences. Selection could impose ratchet-like increases in fitness, but the opposite typically occurs – tissues degenerate with age. How can less fit stem cells dominate their niche? One way to suspend Darwin is through an interesting non-Darwinian phenomenon (32). According to population genetics theory, the role of chance or drift becomes much more important as population size decreases (33, 34). In smaller populations, it becomes increasingly harder to eliminate deleterious mutations or to fix beneficial mutations. Although many parameters influence the balance between chance and selection, generally chance becomes increasingly more important as population sizes slip below one thousand. Crypts stem cell populations are small (<100 stem cells per niche) and therefore chance rather than selection may more determine what types of mutations are fixed (Figure 5). The regions of LOH acquired during human aging did not preferentially fall within regions commonly deleted in CRCs (Figure 4), suggesting random mutation fixation rather than selection.

The numbers of documented somatic CNAs are small, but random mutation fixation due to small niche stem cell numbers may also help explain why neutral passenger mutations are common in carcinomas (35). Many cancers appear later in life, and many alterations found in CRC genomes appear to arise in normal colon before visible tumorigenesis (36, 37). Interestingly, cancer genome mutation frequencies are consistent with relatively normal division and mutation rates (38), suggesting many CRC mutations first accumulate in normal crypts. Importantly, there is a profound lack of evidence for purifying selection in many types of cancer genomes (breast, CRC, pancreatic, glioblastoma, head and neck, ovarian, myeloma, gastric), manifested by the ratio of non-synonymous to synonymous mutations (39, 40). This ratio (dN/dS) is about one and essentially the value expected of random mutation, suggesting that most coding mutations in cancer genomes were not screened by selection. This lack of evidence for somatic mutation selection is curious because the dN/dS ratio is less than one in the human germline (40), indicating that purifying selection normally eliminates many non-synonymous mutations in human populations. The abundance of cancer passenger mutations and the lack of purifying selection may be the legacy of their origins within very small stem cell niches, where mutation selection is nullified by chance fixation.

If non-Darwinian mutation fixation depends on small numbers of niche stem cells, tissues with different niche architectures or dynamics may more often accumulate selective mutations. Whole blood cells originate from multiple hematopoietic stem cell niches that are much more dynamic than crypt niches with respect to physical locations, numbers of stem cells, and migration of stem cells to neighboring niches (28). Instead of a physical subdivision into multiple distinct isolated small stem cell niches, hematopoietic stem cells are not confined to a single niche but normally migrate to new niches. Consistent with a different niche architecture, age-related increases in detectable CNAs in whole blood appear to be more driven by selection because blood CNAs are frequently located in regions commonly altered in hematopoietic malignancies (27). Niche stem cell number size limitations may not apply to the hematopoietic system, and therefore selection may have a greater role in determining whether a mutant stem cell can spread and occupy the majority of hematopoietic stem cell niches.

Random niche mutation fixation is not a fool-proof anti-cancer mechanism because by chance sometimes a potential driver mutation will become fixed instead of discarded, and some somatic mutations may confer selection sufficient to overwhelm random fixation. Many driver mutations such as in APC and TP53 are compatible with normal appearing intestines, and their fixation within a crypt resembles clonal evolution, with a net increase in mutant cells but without visible tumorigenesis. Indeed, some “driver” mutations without immediate apparent selective value may be randomly fixed, expressing their driver functions only in combination with other driver mutations much later in the final tumor or metastasis. Potentially CRCs could result from the random accumulation, in any order, of multiple initially neutral, “driver” mutations in niche stem cells (41). However fewer CRCs would occur with multiple crypt niche stem cells compared to crypts with multiple immortal stem cells (42).

### Non-Darwinian stem cell niche evolution: A testable hypothesis

The hypothesis that stem cell niches harness non-Darwinian evolution can be tested experimentally in model systems by comparing the fates of specific mutations engineered to occur in single isolated stem cells. Mouse crypt niches are likely smaller than human niches, so non-Darwinian effects should be exacerbated. For example, with a mouse model with a mutant *Cre* sporadically reactivated by rare back-mutation, the fixation of an individual intestinal crypt stem cell with a neutral floxed *lacZ* marker (*Rosa26R*) can be compared to the fixation of a stem cell with *Rosa26R* combined with floxed “driver” mutations (43, 44). With Darwinian evolution, driver mutations should confer selective advantages and be fixed much more often, leading to more *lacZ* positive crypts. With non-Darwinian evolution, stem cells with driver mutations should be randomly discarded as often as stem cells with neutral *Rosa26R* mutations, resulting in similar numbers of *lacZ* positive crypts. Predicted differences between niche selection and random fixation are large. With “*N*” niche stem cells (*N* is about 8–12 stem cells per crypt in mice (14, 15), stem cell fixation should be 100% with driver mutation selection, but only 8–12% (“1/*N*”) of stem cells will become fixed with random stem cell loss.

Data with floxed *Kras^G12D^* and *Apc^580S^* driver mutations were more consistent with non-Darwinian evolution or random niche fixation because the numbers of fixed *lacZ* positive mutation events were similar to control mice without the driver mutations (43, 44). Although stem cells with *Kras^G12D^* or *Apc^580S^* mutations did not appeared to be fixed more often in crypt niches, they did confer selective advantages after fixation, manifested by larger patches of mutant crypts due to increased crypt fission. Similar experimental studies can further test whether isolated single niche stem cells with specific somatic mutations are fixed randomly or selectively.

### Crypt stem cell genetic fidelity and non-Darwinian stem cell evolution

Perfect stem cell fidelity would be an “anti-evolution” strategy to never grow old. Aging, or the accumulation of mutations may be inevitable, and the genetic fidelity of human crypt stem cells appears not to be higher than expected of normal cells. Given the inevitability of mutations, the crypt stem cell niche may trade Darwinian for non-Darwinian evolution as a downstream mechanism to manage these mutations (Figure 5). During a lifetime, a critical question is whether deleterious or beneficial mutations are more dangerous to homeostasis. Many “deleterious” somatic mutations may be tolerated by human cells, exemplified by the relatively large numbers of rare but potentially dysfunction mutations in normal human germline genomes (45). The spread of beneficial somatic mutations may pose a greater threat to survival. Niche stem cell turnover may harness a non-Darwinian evolution mechanism (neutral drift) that readily protects against lethal mutations and helps ensure that beneficial mutations that might lead to cancer are often discarded. Given the cooperation needed between multiple cells in mammalian tissues and the dangers of tumorigenesis, an optimal reliable downstream strategy to guard against the unwanted effects of some mutations may be random stem cell fixation in tissues subdivided into very small niches. This non-Darwinian strategy is built into the tissue niche architecture from birth, and can help explain why tissues do not become paradoxically “fitter” with age (Figure 5). This scenario resembles Muller’s ratchet, where asexual division leads to decline (46).

Interesting, non-intuitive phenomenon often emerge at smaller physical dimensions. Multiple, mitotic stem cells in very small niches with non-Darwinian evolution can better explain colon aging and somatic mutation frequencies and spectra compared to Darwinian niche selection. Non-Darwinian evolution may predominate whenever reproducing somatic tissues are physically subdivided into distinct very small isolated compartments. Given the impracticality of human experimental manipulations, the analysis of somatic alterations found in normal human tissues provides a feasible pathway for insights into human stem cell mechanisms. Somatic alterations can reveal much about stem cell life and death, particularly because most mitotic niche stem cell lineages suffer extinction. Newer technologies increasingly provide better methods to detect mutations, and more data on the numbers and types of alterations found in normal human tissues will allow much better inferences on how we age. Small stem cell niches provide a downstream architectural mechanism for randomly discarding many inevitable but unwanted mutations.

## Authors Contribution

Haeyoun Kang helped analyze the data and edit the manuscript. Darryl Shibata supplied the colon crypts, helped analyze the data, and write the manuscript.

## Conflict of Interest Statement

The authors declare that the research was conducted in the absence of any commercial or financial relationships that could be construed as a potential conflict of interest.
